# Climate change and physical activity: ambient temperature and urban trail use in Texas

**DOI:** 10.1007/s00484-022-02302-5

**Published:** 2022-05-27

**Authors:** Kevin Lanza, Julia Gohlke, Suwei Wang, Perry E. Sheffield, Olga Wilhelmi

**Affiliations:** 1 Department of Epidemiology, Human Genetics, and Environmental Sciences, UTHealth School of Public Health in Austin, 1616 Guadalupe St, Suite 6.300, Austin, TX 78701 USA; 2 Department of Population Health Sciences, Virginia Polytechnic Institute and State University, 205 Duck Pond Drive, Blacksburg, VA 24061 USA; 3 Department of Population Health Sciences, Duke University, 215 Morris Street, Durham, NC 27701 USA; 4 Departments of Environmental Medicine and Public Health and Pediatrics, Icahn School of Medicine at Mount Sinai, 1 Gustave L Levy Pl, EMPH Box 1057, New York, NY 10029 USA; 5 Research Applications Laboratory, National Center for Atmospheric Research, P.O. Box 3000, Boulder, CO 80307 USA

**Keywords:** Pedestrian, Cyclist, Adaptation, Extreme heat, Apparent temperature, Humid subtropical climate

## Abstract

Individuals in the USA are insufficiently active, increasing their chronic disease risk. Extreme temperatures may reduce physical activity due to thermal discomfort. Cooler climate studies have suggested climate change may have a net positive effect on physical activity, yet research gaps remain for warmer climates and within-day physical activity patterns. We determined the association between ambient temperatures (contemporary and projected) and urban trail use in a humid subtropical climate. At a trail in Austin, TX, five electronic counters recorded hourly pedestrian and cyclist counts in 2019. Weather data were acquired from World Weather Online. Generalized additive models estimated the association between temperature and trail counts. We then combined the estimated exposure–response relation with weather projections from climate models for intermediate (RCP4.5) and high (RCP8.5) emissions scenarios by NASA NEX-GDDP. From summer to autumn to spring to winter, hourly trail counts shifted from bimodal (mid-morning and early-evening peaks) to one mid-day peak. Pedestrians were more likely to use the trail between 7 and 27 °C (45–81°F) with peak use at 17 °C (63°F) and cyclists between 15 and 33 °C (59–91°F) with peak use at 27 °C (81°F) than at temperature extremes. A net decrease in trail use was estimated by 2041–2060 (RCP4.5: pedestrians =  − 4.5%, cyclists =  − 1.1%; RCP8.5: pedestrians =  − 6.6%, cyclists =  − 1.6%) and 2081–2100 (RCP4.5: pedestrians =  − 7.5%, cyclists =  − 1.9%; RCP8.5: pedestrians =  − 16%, cyclists =  − 4.5%). Results suggest climate change may reduce trail use. We recommend interventions for thermal comfort at settings for physical activity.

## Introduction

Regular physical activity has been linked to reduced risk of all-cause mortality, cardiovascular disease, type 2 diabetes, and several cancers and improved quality of life, mental health, and sleep (Piercy et al. 2018), yet in the USA, only about one-half of adults (54%) in 2018 and one-quarter of adolescents (23%) and children (26%) in 2019 reported meeting the 2008 Physical Activity Guidelines for Americans for aerobic physical activity (US Department of Health and Human Services 2021a, 2021b, 2021c).

To inform the development of built environments that are supportive of physical activity, researchers have aimed to identify the facilitators and barriers of physical activity participation using ecological models, which recognize many multilevel, interacting influences on physical activity (Sallis et al. 2012). Ambient temperature is a potential influence on physical activity that is gaining widespread attention (Bernard et al. 2021) due to climate change causing a rise in overall temperatures and an increase in the intensity, frequency, and duration of heat waves (Anderson and Bell 2011; Smith et al. 2013; Hayhoe et al. 2018). Temperatures outside the thermoneutral zone—the range of ambient temperatures where core body temperature is maintained through regulating skin blood flow (Kingma et al. 2014)—may serve as a barrier to engaging in outdoor physical activity due to thermal discomfort. Furthermore, temperatures in cities are amplified by urban heat islands caused by high amounts of impervious materials, few trees, and waste heat emissions from air conditioning use, motor vehicle use, and other energy-consuming activities (Lanza et al. 2019; Stone Jr et al. 2019).

Three systematic reviews have shown that, in general, ambient temperatures exhibit a positive association with outdoor physical activity (Chan and Ryan 2009; Tucker and Gilliland 2007; Lee et al. 2021); however, most studies took place in temperate climates and assumed constant linear relations between temperature and physical activity (Chan et al. 2006; Belanger et al. 2018; Jones et al. 2017; Duncan et al. 2008; Alahmari et al. 2015). The few studies occurring in subtropical and tropical climates that accounted for potential non-linear relations revealed temperature extremes to associate with a decrease in physical activity (Al-Mohannadi et al. 2016; Baranowski et al. 1993; Chmura et al. 2017; Ermagun et al. 2018; Ridgers et al. 2015; Togo et al. 2005). The temperature-physical activity relationship has not been assessed at the hourly level, which would reveal when and at what temperatures individuals are choosing to be active.

Regarding climate change and physical activity, two studies have used nonparametric statistics to determine the association between ambient temperature and physical activity in projected temperature conditions (as well as historical conditions), with similar results. In a study of adults across the USA between 2002 and 2012, researchers estimated that participation in physical activity increased up to 28–29 °C before declining (Obradovich and Fowler 2017). The authors then coupled the historical model parameters with downscaled data from 21 climate models under a high emissions scenario of greenhouse gases—representative concentration pathway 8.5 (RCP8.5)—to find that most areas of the USA experienced net increases in physical activity in 2050 and 2090 compared to 2002–2012. This nationwide study was limited by relatively coarse temporal resolution (i.e., daily) and spatial resolution (i.e., city level) and use of self-reported physical activity data, which are prone to response bias (Crouch et al. 2018). In a second study on bike share usage in New York City from 2013 to 2017, researchers estimated that as temperatures increased, daily hours and distance ridden significantly increased but then declined above 26–28 °C (Heaney et al. 2019). The authors projected a net increase in bike usage in the period 2040–2069 compared to 1980–2005 under both intermediate (RCP4.5) and high (RCP8.5) greenhouse gas emissions scenarios. Opportunities to advance this New York City–based study include focusing on geographies with different climate and other active transportation modes and infrastructure such as urban trails—wide, paved, and linear infrastructure meant for walking, cycling, and other non-motor vehicle uses and connected to other transportation infrastructure (e.g., sidewalks) to link neighborhoods, community resources, and greenspaces (City of Austin 2021).

Herein, we investigated the associations between ambient temperature—both contemporary and projected—and use of an urban trail by pedestrians and cyclists in a humid subtropical climate. Specifically, we assessed the (1) relations between hourly ambient temperatures and the number of pedestrians and cyclists using an urban trail, and (2) projected impact of climate change on trail use by pedestrians and cyclists from early twenty-first century to mid-century and late century under intermediate and high emissions scenarios of greenhouse gases.

## Materials and methods

### Study setting

This serial cross-sectional study took place across the full year of 2019 at the Ann and Roy Butler Hike-and-Bike Trail, an urban trail located in downtown Austin, TX, that is co-managed by the nonprofit organization The Trail Foundation and the Austin Parks and Recreation Department (Austin Monitor 2021a). The continuous, 16-km-long, 2–6-m-wide, loop trail follows the shoreline of Lady Bird Lake, a 168-hectare reservoir on the Colorado River surrounded by a mix of public greenspace and residential and commercial properties. Eighty-one hectares of greenspace that encircles the lake includes 26 hectares of woodlands, 54 hectares of mowed lawns and open space, areas without vegetation, and Grow Zones—creekside areas that had been regularly mowed but are now recovering naturally dense and diverse native vegetation (The Trail Foundation 2015). The majority of the trail surface is composed of crushed granite except for a few areas of concrete and a 2-km-long boardwalk on the south shore (Austin Monitor 2021b). Public amenities along the trail include viewing areas, art, boat ramps, outdoor exercise equipment, benches, picnic tables, litter receptacles, drinking fountains, and restrooms (The Trail Foundation 2021a).

In 2020, more than 4.5 million unique visits by walkers, runners, and cyclists were recorded on the trail (The Trail Foundation 2021b). Electric bikes traveling under 16 km per hour are permitted on the trail while scooters are only permitted on three of the six bridges that intersect with the trail and cross Lady Bird Lake (The Trail Foundation 2021c). The contiguous trail is publicly accessible year-round at all hours except the boardwalk section, which has a curfew between midnight and 5 am (The Trail Foundation 2021d). To minimize light pollution and its disruption of nature, the trail has no artificial lighting other than low-level lighting at trail segments with low ambient light levels that present a tripping hazard (The Trail Foundation 2021c).

### Measurement of trail users

Over 2019, we recorded hourly counts of pedestrians and cyclists on the Ann and Roy Butler Hike-and-Bike Trail using the Eco-Counter Urban MULTI, an in situ electronic counting system composed of a logger, two sensors, and battery within a 1-m-high, galvanized steel post. The two sensors communicate with each other to determine when a traveler is a pedestrian or a cyclist then registers a count. The electronic counter uses passive-infrared, pyroelectric technology and a high-precision lens to count pedestrians (i.e., walkers, runners) by detecting their body temperature. To measure cyclists, the counter includes a ZELT inductive loop that analyzes the electromagnetic signature of each bicycle using 13 differentiation criteria. The counters detect pedestrians and cyclists within 6 m and can distinguish the direction of travel. The algorithm for detecting travel mode does not include wheelchairs, scooters, skateboards, and other modes; as such, these modes will be categorized as either a pedestrian or cyclist depending on wheel base, metal content, and speed (Eco-Counter 2021a). However, trail use by these other modes is limited due to the crushed granite surface of most of the trail and scooters only being permitted on three bridges intersecting the trail.

**Fig. 1:**
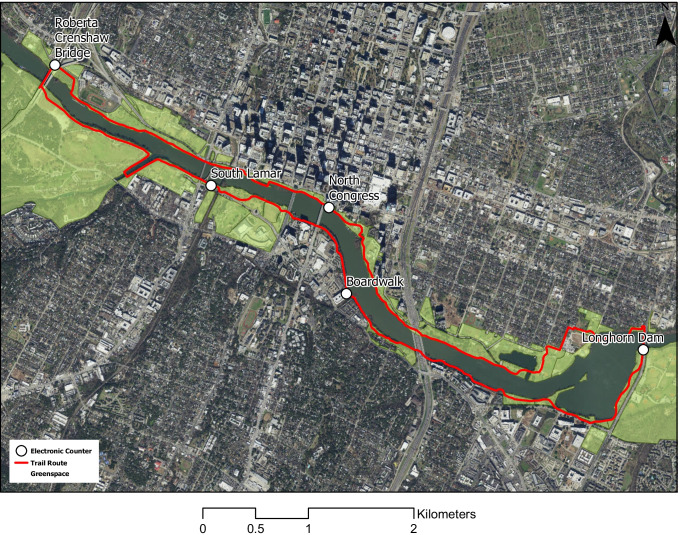
Electronic counters measuring pedestrian and cyclist counts on the Ann and Roy Butler Hike-and-Bike Trail at Lady Bird Lake by summing the counts of each travel mode in both directions per counter

The Urban Trails Program of the City of Austin Public Works Department maintains five counters spaced evenly throughout the trail (Fig. 1) (The Trail Foundation 2021b). The first counter installed along the trail was at the Boardwalk in May 2015, followed by a counter at Roberta Crenshaw Bridge in February 2016, and lastly counters at Longhorn Dam, North Congress, and South Lamar in March 2018. We focused on counter data from 2019 since this year was prior to the COVID-19 pandemic and its potential impact on trail use, and had the most complete data for the five electronic counters. The counter at Roberta Crenshaw Bridge recorded data every hour in 2019, while downtime between battery replacements for the other four counters resulted in below 100% completeness of 2019 hourly data (i.e., Boardwalk = 62%, Longhorn Dam = 96%, North Congress = 78%, South Lamar = 98%). We calculated two variables for hourly counts of pedestrians and cyclists by summing the counts of each travel mode in both directions per counter.

### Measurement of observed weather conditions

Ambient air temperature, ambient apparent temperature (i.e., heat stress index of air temperature, relative humidity, wind speed), and amount of rainfall in 2019 originated from Eco-Counter’s Eco-Visio Weather Module, which pulls historical weather data from World Weather Online to provide average hourly weather conditions specific to each Eco-Counter Urban MULTI electronic counter (Eco-Counter 2021b). The Eco-Visio Weather Module permits historical weather data to be immediately available alongside pedestrian and cyclist count data from each counter. World Weather Online generates aggregated weather data from a range of sources (e.g., in situ weather stations, global weather satellite imagery) and data are reviewed for accuracy by meteorologists on staff (World Weather Online 2021). No weather stations were located directly along the trail. Rather than measure trail-specific meteorological data, the intent was to measure data from the built up surroundings since this is where individuals are potentially coming from (and pulling weather data from) when deciding whether to visit the trail. Hourly data from World Weather Online were similar to those recorded from the nearest weather station of the Global Historical Climatology Network at Austin Camp Mabry: in 2019, datasets matched 92% of the time on whether it rained each hour and 79% of the time, hourly ambient air temperature values between datasets were within ± 3 °C (National Oceanic and Atmospheric Administration 2021). Differences in rainfall and temperature may be partly related to different microclimates modeled by World Weather Online at counter locations and directly measured at the Camp Mabry location.

### Measurement of projected weather conditions

We used the publicly available, no-cost NASA Earth Exchange Global Daily Downscaled Projections (NEX-GDDP) dataset to estimate future ambient temperature and rainfall conditions (National Aeronautics and Space Administration 2021), per previous studies (Heaney et al. 2019; Yang and Li 2021; Huang et al. 2018; Obradovich and Fowler 2017). Covering the globe from 1950 to 2100, the NEX-GDDP dataset comprises downscaled (25-km spatial resolution) and bias-corrected output from 21 climate models—derived from the general circulation model runs conducted under the Coupled Model Intercomparison Project Phase 5 (Taylor et al. 2012)—for greenhouse gas emissions scenarios RCP4.5 and RCP8.5 (Thrasher et al. 2012). The dataset does not include downscaled projections for RCP2.6 and RCP8.5, the two other pathways used in the Fifth Assessment Report of the United Nations Intergovernmental Panel on Climate Change.

For the 25-km grid cell containing the Ann and Roy Butler Hike-and-Bike Trail, we calculated daily averages of maximum air temperature and rainfall amount across the 21 models under both RCP4.5 and RCP8.5 at three time periods: early century (2006–2025), mid-century (2041–2060), and late century (2081–2100). We included both mid-century and late century because of the potential for different public health implications and solutions at these time horizons, and chose to analyze both RCP4.5 and RCP8.5 due to mixed scientific opinion on which scenario most likely reflects the future (Hausfather and Peters 2020; Schwalm et al. 2020).

### Statistical analyses

We first calculated the mean and standard deviation for observations (i.e., pedestrians and cyclists using the trail on weekdays and weekends, ambient temperature, rainfall) in 2019 and for climate projections (i.e., ambient temperature, rainfall) for early century, mid-century, and late century under RCP4.5 and RCP8.5. We presented these summary statistics of the observations in 2019 at the daily level rather than hourly for consistency with projections. Along with summary statistics for the full year of 2019, we stratified by spring (March 1–May 31), summer (June 1–August 31), autumn (September 1–November 30), and winter (December 1–February 28). We then calculated average counts of pedestrians and cyclists per hour for each season as well as for a subset of summer days (*n* = 10)—called herein “summer heat days”—when daily maximum air temperature was greater than or equal to the 90th percentile (37.8 °C), per previous research (Lanza et al. 2020).

To test the associations between hourly ambient air temperature and pedestrian and cyclists counts, we used Poisson nonparametric generalized additive models—with electronic counters as a random effect and including rainfall (hourly millimeter) and weekends (1 = weekend, 0 = not weekend) as covariates—to test for associations with physical activity levels (Zhao et al. 2019; Lanza et al. 2021b, 2020). Generalized additive models allow for non-linear relations between independent variables and the outcome and have been applied to assess non-linear associations between ambient temperature or air pollution and health outcomes (Halonen et al. 2016; Heaney et al. 2019; Muggeo and Hajat 2009; Winckelmans et al. 2015). We performed separate models for pedestrians and cyclists for the full year of 2019 and by season, and also ran models using ambient apparent temperature rather than air temperature. We reported exponentiated independent variable coefficients as risk ratios.

Next, we predicted the impact of climate change on trail use by combining the estimated exposure–response function—from the generalized additive model results mentioned above—with the daily projections of temperature and rainfall for early century, mid-century, and late century under RCP4.5 and RCP8.5. From the predicted daily output, we calculated the percent change in pedestrians and cyclists using the trail each month from early century to mid-century and late century under RCP4.5 and RCP8.5. Statistical analyses were completed in R (v.1.4.1717) using the mgcv (v.1.8–36) package. The datasets generated and analyzed during the current study are available in the Mendeley Data repository (10.17632/23jjc7z3n8.1) (Lanza 2022).

## Results

For daily averages across the five counters in 2019 (Table 1), air temperatures ranged from 12 °C (minimum = 8 °C, maximum = 16 °C) in the winter to 30 °C (minimum = 25 °C, maximum = 35 °C) in the summer. Spring and autumn days exhibited similar temperatures of 20 °C and 21 °C, respectively. Summer had the least amount of daily rainfall (2 mm) yet had the most days with any rainfall (29%). Winter days were least likely to have any rainfall (12%). Throughout the year, more pedestrians used the trail daily than cyclists. There were more pedestrians and cyclists using the trail on weekends than on weekdays throughout the year. More pedestrians used the trail on weekdays during the spring (1,945) and winter (1,852) compared to summer (1,243) and autumn (1,190). On weekends, the highest daily count of pedestrians occurred in the winter (3,668), followed by spring (2,893); autumn (2,467); and summer (1,900). Summer had the most cyclists each day (weekday = 481, weekend = 655) and winter had the least (weekday = 254, weekend = 441). 

For daily averages for each counter in 2019 (Appendix Table 3), the counter at Longhorn Dam had the lowest counts of pedestrians and cyclists on weekdays (pedestrians = 475, cyclists = 237) and weekends (pedestrians = 1,026; cyclists = 354). The counter at North Congress recorded the most pedestrians (weekday = 2,131; weekend = 3,288). The counter at the Boardwalk had the most cyclists (weekday = 663, weekend = 904).

**Table 1:** Summary statistics of daily observed weather conditions and trail use in 2019 across electronic counters

	Full year 2019^a^	Spring 2019^a^	Summer 2019^a^	Autumn 2019^a^	Winter 2019^a^
Variables^b^	Mean ± Std. Dev	Mean ± Std. Dev	Mean ± Std. Dev	Mean ± Std. Dev	Mean ± Std. Dev
Mean air temperature (°C)	21 ± 8^c^	20 ± 5	30 ± 2	21 ± 8	12 ± 4
Min. air temperature (°C)	16 ± 8^c^	16 ± 6	25 ± 2	17 ± 8	8 ± 4
Max. air temperature (°C)	26 ± 9^c^	25 ± 6	35 ± 3	26 ± 8	16 ± 5
Rain (mm)^d^	2 ± 9	4 ± 11	2 ± 3	2 ± 8	2 ± 12
Pedestrians—weekday (#)	1,560 ± 652	1,949 ± 728	1,243 ± 373	1,190 ± 460	1,852 ± 593
Cyclists—weekday (#)	376 ± 152	401 ± 156	481 ± 75	367 ± 149	254 ± 116
Pedestrians—weekend (#)	2,715 ± 1,062	2,893 ± 798	1,900 ± 389	2,467 ± 966	3,668 ± 1,115
Cyclists—weekend (#)	555 ± 179	524 ± 246	655 ± 72	589 ± 65	441 ± 194

^a^Percentage of days with missing hourly data from more than one electronic counter: full year = 5%, spring = 10%, summer = 0%, autumn = 0%, winter = 11%

^b^All variables are average daily values across the five electronic counters

^c^Air temperature (°F): mean = 70 ± 14, min. = 61 ± 14, max. = 79 ± 16

^d^Percentage of days with any rainfall: full year = 23%, spring = 27%, summer = 29%, autumn = 22%, winter = 12%

**Fig. 2:**
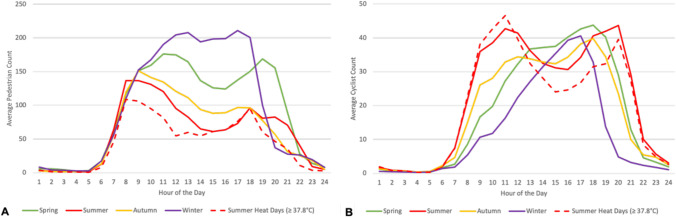
Average trail use by **a** pedestrians and **b** cyclists per hour in 2019 for each season and summer heat days where daily maximum air temperature was greater than or equal to the 90th percentile (37.8 °C)

From summer to autumn to spring to winter, the distribution of the average number of pedestrians and cyclists each hour across all counters shifted from bimodal (with peak use in mid-morning and early-evening hours) to a single mid-day peak (Fig. 2). For pedestrians, hourly peaks of trail use increased from summer to autumn to spring to winter. The highest pedestrian count occurred at 17:00 during the winter (*n* = 211). Summer heat days had the lowest pedestrian counts across most hours compared to those on all other days throughout the year, with almost a four times lower (3.8) pedestrian count at noon compared to the same time on winter days. For cyclists, trail use shifted to later times in the morning from summer to autumn to spring to winter, and to later times in the evening from winter to autumn to spring to summer. Seasonally, the highest cyclist count occurred at 18:00 during the spring and 20:00 during the summer (*n* = 44); however, when looking at a subset of summer days, 11:00 on days when air temperatures were greater than or equal to 37.8 °C had the highest cyclist count (*n* = 47) compared to hours on all other days throughout the year.

**Fig. 3:**
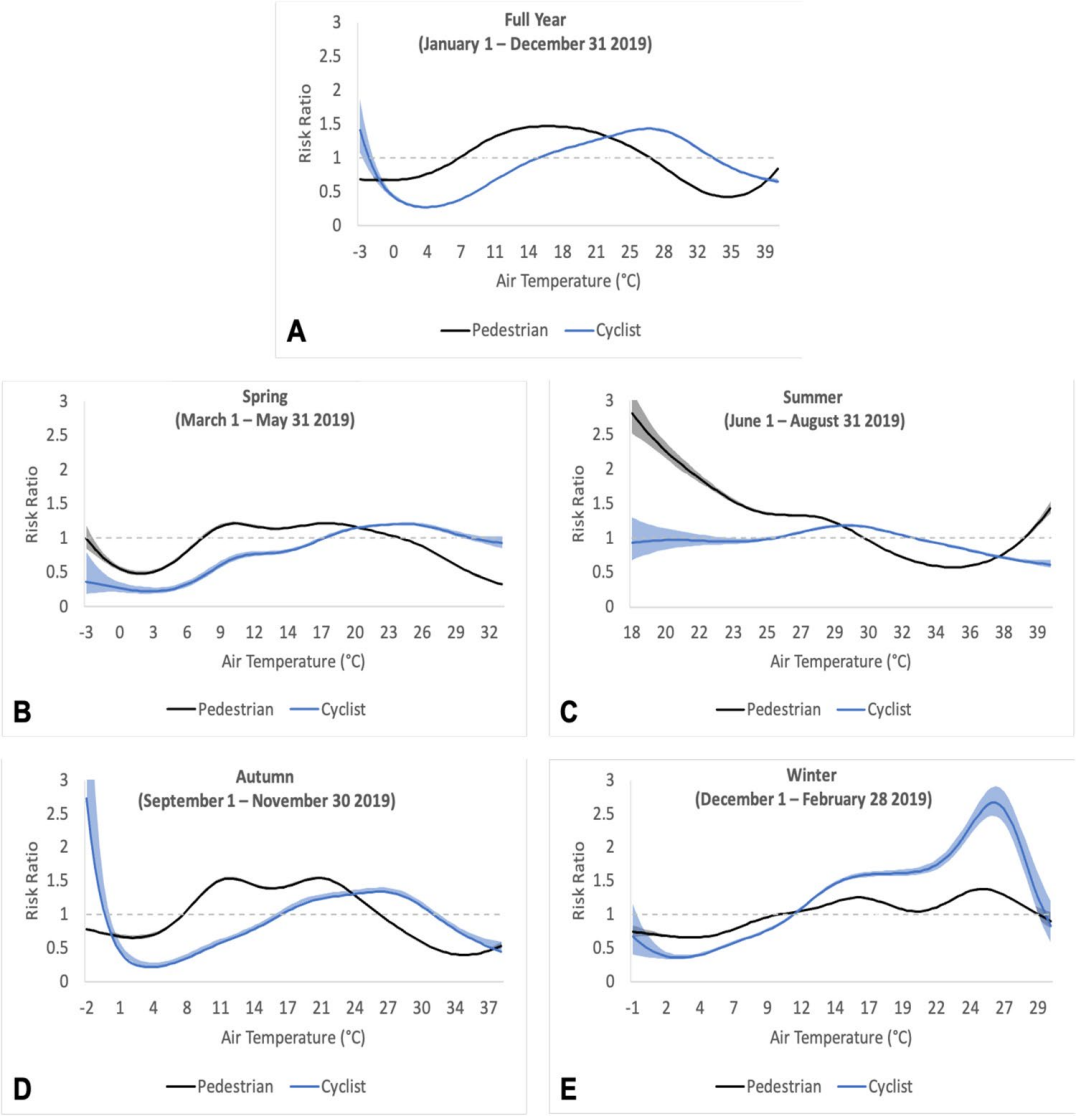
Model output for relations between air temperature and trail use by pedestrians and cyclists in 2019 during the **a** full year, **b** spring, **c** summer, **d** autumn, and **e** winter. Shading along the curve represents 95% confidence intervals

Holding all other covariates constant, pedestrians and cyclists were more likely to use the trail at ambient air temperature ranges of 7–27 °C and 15–33 °C, respectively, than at temperature extremes: risk ratios were equal to 1 at 7 °C and 27 °C for pedestrians and 15 °C and 33 °C for cyclists (Fig. 3a). For pedestrians, peak trail use occurred at 17 °C: 1.5 (95% CI = 1.5–1.5) times the number of pedestrians used the trail at 17 °C than at 7 °C and 27 °C. For cyclists, peak trail use occurred at 27 °C: 1.4 (95% CI = 1.4–1.5) times the number of cyclists used the trail at 27 °C than at 15 °C and 33 °C.

In spring (Fig. 3b), pedestrians were more likely to use the trail between 7 and 24 °C with peak use at 18 °C, and cyclists between 18 and 30 °C with peak use at 25 °C. In summer (Fig. 3c), pedestrians were more likely to use the trail until 30 °C with peak use at 18 °C, and cyclists between 26 and 33 °C with peak use at 29 °C. In autumn (Fig. 3d), pedestrians were more likely to use the trail between 7 and 26 °C with peak use at 20 °C, and cyclists between 17 and 32 °C with peak use at 26 °C. In winter (Fig. 3e), pedestrians were more likely to use the trail between 10 and 29 °C with peak use at 25 °C, and cyclists between 11 and 30 °C with peak use at 26 °C . Apparent temperature exhibited similar associations with trail use by pedestrians and cyclists as air temperature (Appendix Fig. 5).

Compared to daily maximum temperatures averaged across the five counters in 2019 (26 °C) (Table 1), projections for daily maximum temperatures averaged across the 21 climate models under RCP4.5 and RCP8.5 were higher in early century (RCP4.5 = 27 °C, RCP8.5 = 27 °C) (Table 2). From early to mid- to late century, projections for average daily maximum temperature increased 1 °C each period under RCP4.5 (early = 27 °C, mid = 28 °C, late = 29 °C) and two-degrees Celsius under RCP8.5 (early = 27 °C, mid = 29 °C, late = 31 °C). During summer in late century, average daily projections for maximum temperatures were 37 °C under RCP4.5 and 40 °C under RCP8.5. During winter in late century, average daily projections for minimum temperatures were 20 °C under RCP4.5 and 22 °C under RCP8.5. Projections for average daily rainfall in early century (RCP4.5 = 2.2 mm, RCP8.5 = 2.1 mm) were slightly lower than observed in 2019 (2.4 mm) and remained relatively unchanged by late century (RCP4.5 = 2.1 mm, RCP8.5 = 2.1 mm).

**Table 2:** Summary statistics of projected daily weather conditions during early, mid-, and late twenty-first century under RCP4.5 and RCP8.5

	Full year	Spring	Summer	Autumn	Winter
Variables^a^	Mean ± Std. Dev	Mean ± Std. Dev	Mean ± Std. Dev	Mean ± Std. Dev	Mean ± Std. Dev
*RCP4.5 early (2006–2025)*					
Max. air temperature (°C)	27 ± 7^b^	27 ± 3	36 ± 1	28 ± 5	18 ± 1
Rain (mm)	2.2 ± 1.1	2.5 ± 1.3	2.0 ± 1.2	2.5 ± 1.1	1.7 ± 0.7
*RCP4.5 mid (2041–2060)*					
Max. air temperature (°C)	28 ± 7^b^	28 ± 3	37 ± 1	30 ± 5	19 ± 1
Rain (mm)	2.1 ± 1.1	2.4 ± 1.2	2.0 ± 1.1	2.5 ± 1.1	1.7 ± 0.7
*RCP4.5 late (2081–2100)*					
Max. air temperature (°C)	29 ± 7^b^	29 ± 4	37 ± 1	30 ± 5	20 ± 1
Rain (mm)	2.1 ± 1.1	2.4 ± 1.3	2.0 ± 1.2	2.5 ± 1.2	1.7 ± 0.7
*RCP8.5 early (2006–2025)*					
Max. air temperature (°C)	27 ± 7^b^	27 ± 3	36 ± 1	28 ± 5	18 ± 1
Rain (mm)	2.1 ± 1.2	2.5 ± 1.3	2.0 ± 1.3	2.5 ± 1.1	1.6 ± 0.6
*RCP8.5 mid (2041–2060)*					
Max. air temperature (°C)	29 ± 7^b^	29 ± 3	37 ± 1	30 ± 5	20 ± 1
Rain (mm)	2.1 ± 1.2	2.4 ± 1.3	2.0 ± 1.3	2.4 ± 1.1	1.6 ± 0.7
*RCP8.5 late (2081–2100)*					
Max. air temperature (°C)	31 ± 7^b^	31 ± 4	40 ± 1	33 ± 5	22 ± 1
Rain (mm)	2.0 ± 1.1	2.1 ± 1.1	1.8 ± 1.1	2.5 ± 1.2	1.5 ± 0.6

^a^All variables are average daily values across the 21 climate models per representative concentration pathway

^b^Max. air temperature (°F): RCP4.5 early = 81 ± 13, RCP4.5 mid = 82 ± 13, RCP4.5 late = 84 ± 13, RCP8.5 early = 81 ± 13, RCP8.5 mid = 84 ± 13, RCP8.5 late = 88 ± 13

**Fig. 4:**
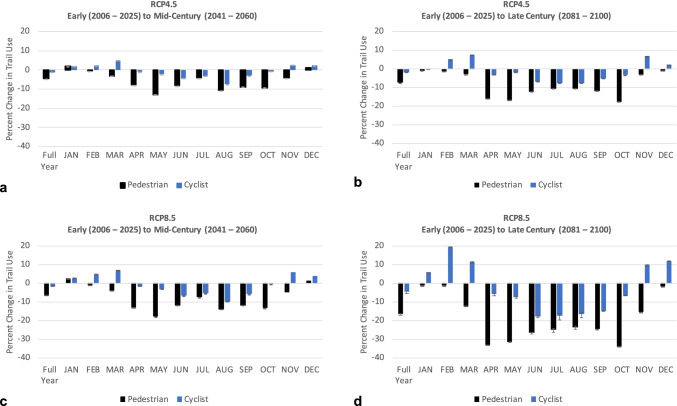
Percent change in trail use **a** early to mid-century under RCP4.5, **b** early to late century under RCP4.5, **c** early to mid-century under RCP8.5, and **d** early to late century under RCP8.5

Under RCP4.5 from early to mid-century (Fig. 4a), we predicted that overall use of the trail by pedestrians and cyclists would decrease 4.5% (95% CI = 4.5–4.6%) and 1.1% (95% CI = 1.1–1.2%), respectively. By late century under RCP4.5 (Fig. 4b), overall use would decrease 7.5% (95% CI = 7.4–7.6%) for pedestrians and 1.9% (95% CI = 1.8–2.1%) for cyclists. Under RCP8.5 from early to mid-century (Fig. 4c), overall use of the trail by pedestrians and cyclists would decrease 6.6% (95% CI = 6.5–6.7%) and 1.6% (95% CI = 1.5–1.7%), respectively. By late century under RCP8.5 (Fig. 4d), overall use would decrease 16.7% (95% CI = 16.5–17.0%) for pedestrians and 4.5% (95% CI = 4.1–5.4%) for cyclists. By mid- and late century under RCP4.5 and RCP8.5, we projected a decrease in both pedestrians and cyclists using the trail from April through October. We estimated an increase in the percentage of pedestrians from early to mid-century in January and February, which changed to a decrease by late century. Lastly, we projected an increase in cyclists using the trail by mid- and late century in February, March, November, and December.

## Discussion

At the Ann and Roy Butler Hike-and-Bike Trail in Austin, TX, our major findings were that (1) pedestrians and cyclist counts had comparable within-day patterns in 2019 that shifted with season; (2) pedestrians were more likely to use the trail between 7 and 27 °C (45–81°F) with peak use at 17 °C (63°F) and cyclists between 15 and 33 °C (59–91°F) with peak use at 27 °C (81°F), in 2019; and (3) under two greenhouse gas emissions scenarios, we projected that overall trail use would decrease by mid-century (RCP4.5: pedestrians =  − 4.5%, cyclists =  − 1.1%; RCP8.5: pedestrians =  − 6.6%, cyclists =  − 1.6%) and continue to decrease by late century (RCP4.5: pedestrians =  − 7.5%, cyclists =  − 1.9%; RCP8.5: pedestrians =  − 16.7%, cyclists =  − 4.5%).

In this study, the electronic counters along the trail recorded higher overall counts of pedestrians than cyclists, similar to previous studies (Zheng et al. 2020; Beitel et al. 2018). Walking is a more readily available mode of transportation than cycling, in terms of cost and ability. Our finding that trail use was higher on weekends than weekdays adds to the mixed literature (Zhao et al. 2019; Boufous et al. 2018). The relative lack of connectivity between residences and workplaces may prevent the trail from being a viable commute option from Monday through Friday, and the recreational nature of the loop trail (e.g., viewing areas, benches, picnic tables, outdoor exercise equipment) may lead to more visits on weekends when individuals have more discretionary time. Diurnal profiles of pedestrians and cyclists on the trail roughly corroborate findings from the 2017 National Household Travel Survey, which reported that across the USA, walking and biking for exercise, recreation, and to access destinations took place steadily during daylight hours over the year, with four peaks at 8:00, 12:00, 15:00, and 18:00 (US Federal Highway Administration 2020). Our diurnal profile adds further detail to the 2017 National Household Travel Survey by providing seasonal and mode-specific data.

Our finding that trail use by pedestrians in 2019 declined at 27 °C corroborates other studies that focused on the temperature-physical activity relationship in similar climates. Researchers observed significant differences (*p* ≤ 0.001) in the number of sprints by adult soccer players in Rio de Janeiro when air temperature was above 28 °C (31 ± 9 sprints) compared to below 22 °C (40 ± 11 sprints) (Chmura et al. 2017). Another study revealed that pedometer-measured step counts and aerobic step counts (i.e., more than 60 steps per minute or more than 10-min continuous activity) declined when wet bulb globe temperature—an index of air temperature, relative humidity, wind speed, and solar radiation—exceeded 28 °C in Qatar (Al-Mohannadi et al. 2016). Our finding that peak trail use by pedestrians occurred at 17 °C equaled the mean outdoor temperature when step counts peaked in a study of older adults in Nakanjo, Japan (Togo et al. 2005), which shares a humid subtropical climate with Austin.

A nationwide study of the USA revealed general physical activity of adults declined when mean monthly maximum air temperature exceeded 28–29 °C (Obradovich and Fowler 2017), which is between the two upper temperature thresholds for pedestrians (27 °C) and cyclists (33 °C) estimated in our study. Additionally, the nationwide study projected a net decrease in general physical activity of adults in Austin by 2050 and 2099 under RCP8.5 (Obradovich and Fowler 2017), similar to our findings of a net decrease in trail users by mid-century and late century under RCP4.5 and RCP8.5. The authors estimated physical activity to decrease 0–20% in May–October and increase 0–10% in November–April, comparable to our projections of less pedestrians and cyclists using the trail in April–October and more trail use by pedestrians in January–February and by cyclists in November, December, February, and March.

Our finding that trail use by cyclists in 2019 declined at 33 °C differed from another study that estimated a decline in bike share usage in New York City when daily maximum air temperature exceeded 26–28 °C (Heaney et al. 2019). These differences may be due to acclimatization—beneficial physiological adaptations from repeated exposure to high ambient temperature conditions that allow the body to better cope with heat stress. Cyclists in Austin may be acclimatized to higher temperatures than those in New York City: mean maximum temperature normals (1991–2020) recorded from weather stations at Austin-Bergstrom International Airport and John F. Kennedy International Airport are substantively different throughout the year (Austin = 27 °C, New York City = 16 °C) and in August (Austin = 36 °C, New York City = 28 °C) (US National Weather Service 2021). The climate differences between Austin and New York City may explain the different projected cyclist counts in these two locations: we found that overall trail use by cyclists would decrease by mid-century (RCP4.5: − 1.1%, RCP8.5: =  − 1.6%) and late century (RCP4.5: =  − 1.9%, RCP8.5: =  − 4.5%) in Austin while the study in New York City projected a net increase in average annual hours ridden (RCP4.5 = 2.6 ± 1.3%, RCP8.5 = 3.1 ± 1.6%) and distance ridden (RCP4.5 = 0.59 ± 0.18%, RCP8.5 = 4.5 ± 1.6%) by 2040–2069 (Heaney et al. 2019). Additionally, the authors had estimated a decrease in total hours ridden (RCP4.5 =  − 2.9 ± 0.13%, RCP8.5 =  − 3.1 ± 1.6%) and distance ridden (RCP4.5 =  − 0.6 ± 0.2%, RCP8.5 =  − 0.9 ± 0.3%) in June–August in New York City. Lastly, observed differences in temperature associations between our study and the New York City–based study may be ascribed to different proportions of individuals using the trail and bike share program, respectively, for commuting and recreation.

We posit the estimated decrease in trail use in Austin during contemporary ambient temperature extremes and projected net decrease in trail use with climate change are attributed to thermal discomfort of individuals at elevated temperatures serving as a barrier to individuals choosing to use the trail. Physiologically, engaging in physical activity in high ambient temperature conditions stresses an individual’s thermoregulatory system (Cramer and Jay 2016). Individuals may modify their behavior to avoid experiences where they would be prone to overheating. Our hypothesis is based on psychological adaptation, a model that—instead of assuming a stagnant relationship between physiological strain, thermal sensation, and thermal discomfort—proposes a feedback loop where an individual’s past and current thermal experiences affect their perception and reaction to stimuli (Brager and De Dear 1998). We suspect that cyclists were more likely to use the trail at higher temperatures than pedestrians (33 °C versus 27 °C) in our study because cyclists generate more wind (i.e., apparent wind) than pedestrians, which can assist with shedding body heat through evaporation of sweat. For this same reason, we suspect that pedestrians were more likely to use the trail at lower temperatures than cyclists (7 °C versus 15 °C) because apparent wind generated by cyclists would cause them to be uncomfortably cold. For diurnal profiles of physical activity, seasonal differences in hourly average trail counts may be related to individuals electing to engage in physical activity when most thermally comfortable during daylight hours: morning and evening in warmer months when temperatures are lower and mid-day in cooler months when temperatures are higher.

Based on study results, we recommend that municipalities consider programmatic and environmental interventions for thermal comfort at settings intended for physical activity. Events on trails and surrounding greenspace such as fitness classes, group bike rides, and sports practices and competitions can be scheduled during hours of the day—specific to season and travel mode—that may maximize participation and minimize thermal discomfort. Cities in warm climates can work alongside academic and civic partners to develop “Cool Corridors,” defined as a spatially continuous network of physical activity infrastructure outdoors and indoors (e.g., sidewalks, trails, open spaces, pools, gyms) combined with urban heat management strategies (e.g., tree planting, installation of artificial shade structures, construction of naturally ventilated spaces). For instance, researchers revealed that heat index—a combination term of air temperature and relative humidity—was 6 °C cooler under tree shade than unshaded areas in a school park in Austin, TX, and a larger percentage of children (64% versus 46%) were observed under tree shade in higher ambient temperatures than lower temperatures (Lanza et al. 2021a). With its surrounding greenspace and relatively high tree canopy coverage, the Ann and Roy Butler Hike-and-Bike Trail may qualify as a Cool Corridor, potentially protecting against larger reductions in trail use experienced in areas without cooling features during high ambient temperatures. Cool Corridors can be a community health resource that induces physical activity while moderating urban heat islands, which are increasing ambient temperatures in cities alongside climate change to dangerous levels (Leal Filho et al. 2018). By reducing ambient heat exposure, cities can minimize the risk of exertional heat illness from physical activity participation. Heat management strategies of Cool Corridors can have a flexible design (e.g., retractable shade structures) so they do not lower ambient temperatures outside the thermoneutral zone for physical activity during colder months and in cooler climates.

We note a few limitations of this study. First, our focus on one urban trail at one location in one climate region may limit the generalizability of findings to other contexts. Second, ecological models acknowledge the many influences on physical activity behavior including individual characteristics (e.g., age, sex) and environmental characteristics (e.g., tree canopy) (Sallis et al. 2012), yet our regression models predicting the temperature-trail use relationship did not adjust for several of these potential covariates. Similarly, our investigations of the relations between temperature—both contemporary and projected—and trail use did not include a variable for solar radiation, which can influence thermal comfort (Hodder and Parsons 2007), since these data were not available from World Weather Online and the NEX-GDDP dataset. Third, we did not have data on physical activity intensity, frequency, or duration of counted pedestrians and cyclists, all of which influence the level of health benefits from physical activity (Piercy et al. 2018). Fourth, electronic counters categorized all trail users into pedestrians and cyclists; future studies can assess how ambient temperatures affect other travel modes. Lastly, individuals on the loop trail may have traveled past multiple in situ electronic counters, but we were unable to measure whether we counted the same individuals more than once each hour.

## Conclusions

We determined the impact of climate change on trail use in Austin, TX, by averaging weather data projected for early, mid-, and late century from climate models under intermediate and high greenhouse gas emissions scenarios, and then coupling these data with an exposure–response relationship estimated using observed counts of trail users and weather conditions in 2019. We found that pedestrians and cyclists were less likely to use the trail at temperature extremes, and pedestrian use decreased at a lower temperature than that found for cyclists. Compared to early century, we projected net decreases in trail use by mid- and late century under both greenhouse gas emissions scenarios. Estimated increases in trail use from projected warming in several autumn and winter months were more than offset by estimated decreases in trail use from projected warming from April through October. With warming from continued greenhouse gas emissions and urban development, cities might consider combining heat management strategies with physical activity interventions to potentially facilitate comfortable and safe physical activity for health and wellbeing.

## Figures and Tables

**Fig. 5:**
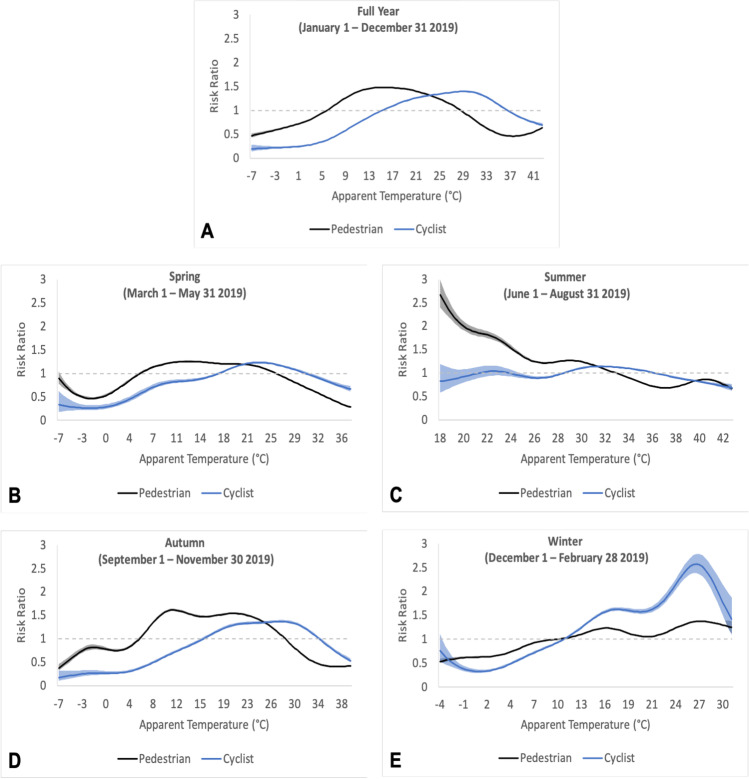
Model output for relations between apparent temperature and trail use by pedestrians and cyclists in 2019 during the **a** full year, **b** spring, **c** summer, **d** autumn, and **e** winter. Shading along the curve represents 95% confidence intervals

## Appendix

**Table 3:** Summary statistics of daily observed weather conditions and trail use in 2019 by electronic counter

	Boardwalk^1^	Crenshaw Bridge^1^	Longhorn Dam^1^	North Congress^1^	South Lamar^1^
Variables^2^	Mean ± Std. Dev	Mean ± Std. Dev	Mean ± Std. Dev	Mean ± Std. Dev	Mean ± Std. Dev
Mean air temperature (°C)	21 ± 8	21 ± 8	21 ± 8	20 ± 8	20 ± 8
Min. air temperature (°C)	17 ± 8	17 ± 8	17 ± 8	15 ± 8	15 ± 8
Max. air temperature (°C)	26 ± 9	26 ± 9	26 ± 9	25 ± 8	25 ± 8
Rain (mm)	3 ± 12	3 ± 12	3 ± 12	2 ± 6	2 ± 6
Pedestrians—weekday (#)	1,813 ± 673	1,770 ± 1,336	476 ± 230	2,131 ± 1,149	1,613 ± 795
Cyclists—weekday (#)	663 ± 212	274 ± 107	237 ± 133	552 ± 187	342 ± 154
Pedestrians—weekend (#)	2,763 ± 1,049	3,200 ± 2,516	1,026 ± 420	3,288 ± 1,086	3,012 ± 1,329
Cyclists—weekend (#)	904 ± 164	421 ± 130	354 ± 174	764 ± 244	543 ± 185

^a^Percentage of days with missing hourly data: Boardwalk = 38%, Crenshaw Bridge = 0%, Longhorn Dam = 4%, North Congress = 22%, South Lamar = 1%

^b^All variables are average daily values for each of the five electronic counters
